# Norovirus diarrhea is significantly associated with higher counts of fecal histo-blood group antigen expressing *Enterobacter cloacae* among black South African infants

**DOI:** 10.1080/19490976.2021.1979876

**Published:** 2021-09-29

**Authors:** Cliff A Magwira, Duncan Steele, ML Seheri

**Affiliations:** Diarrheal Pathogens Research Unit (Dpru), Department of Medical Virology, Sefako Makgatho Health Sciences University, Pretoria, South Africa

**Keywords:** Diarrhea, norovirus infection, histo-blood group antigens, *E. cloacae*, abundance

## Abstract

The study tested the hypothesis that harboring high levels of histo-blood group antigen-expressing *Enerobactero cloacae* is a risk factor for norovirus diarrhea. The fecal *E. cloacae* abundance in diarrheic norovirus positive (DNP), non-diarrheic norovirus negative (NDNN), diarrhea norovirus negative (DNN), and non-diarrhea norovirus positive (NDNP) infants was determined by qPCR, and the risk of norovirus diarrhea was assessed by logistical regression. DNP infants contained significantly higher counts of *E. cloacae* than NDNN and DNN infants, *p* = .0294, and 0.0001, respectively. The risk of norovirus diarrhea was significantly high in infants with higher counts of *E. cloacae* than those with lower counts, *p* = .009. Harboring higher counts of *E. cloacae* is a risk factor for norovirus diarrhea.

## To the editor

Human noroviruses (HNoVs) are the leading cause of sporadic cases and outbreaks of viral gastroenteritis globally, and are responsible for about 700 million infections and 200,000 deaths annually^1^. They initiate infection by attaching to the receptor of various target cells,^2,3^ where they undergo a replication cycle. Histo-blood group antigens (HBGA), expressed by the host, are recognized as susceptibility and cell attachment factors for norovirus, and promote their infection.^4,5^ Emerging evidence suggest that intestinal bacteria also promote efficient norovirus infection of the gastrointestinal tract.^6,7^ For instance, antibiotic-treated mice have been shown to display reduced acute virus titers in the distal ileum, mesenteric lymph nodes and colon compared to the control mice, indicating a decrease in viral replication in vivo following antibiotic treatment.^6^ Similarly, germ-free mice have been shown to shed reduced amount of infectious norovirus in the feces compared to their colonized counterparts.^8^

The underlying mechanism through which intestinal bacteria facilitate norovirus infection is not fully understood. However, intestinal bacteria such as *Enterobacter cloacae* expressing H-type HBGA on its surface has been shown to enhance viral attachment and infection in lymphocytes.^6^ When incubated with *E. cloacae*, the HuNoV GII.4 Sydney virus was able to infect B cells in vitro, whereas incubation with *E. coli* lacking H antigen could not. In addition, the filtration of virus-positive stool sample to remove commensal bacteria has been shown to reduce virus infectivity, whereas the supplementation of filtered stool sample with HBGA-expressing bacteria restored the infectivity. These findings indicate that human noroviruses interact with enteric bacteria by binding to the HBGAs expressed by bacteria, facilitate productive attachment and infection of target cells.^6^

Several other Gram-negative bacteria including *Enterobacter aerogenes, Shigella flexneri, Escherichia fergusonii, E. coli* LMG8223, *E. coli* LFMFP861 have been isolated from human feces and shown to express HBGAs.^9–11^ It is possible that children susceptible to norovirus infection harbor higher counts of intestinal HBGA-expressing bacteria compared to those resistant to the infection. Higher counts of HBGA-expressing bacteria mean more norovirus particles will be facilitated to attach and replicate in the target epithelial cells, resulting in an efficient infection. However, despite their significance, little is known about the abundances of HBGA-expressing bacteria and their association with norovirus infections. This prompted the study to investigate the abundance of *E. cloacae* in fecal samples and their association with norovirus infection among black South African infants. Understanding and identifying specific bacterial strains that promote norovirus infection is crucial in the design and development of oral norovirus vaccines. Such vaccines could be co-administered with probiotic bacterial strain expressing H-type HBGA that could promote attachment and replication of attenuated noroviruses to the host cells.

In this study, fecal samples from a total of 260 infants under the age of one, who reported at a healthcare clinic north of Pretoria, South Africa for diarrhea were assayed for norovirus. Of these, 25 diarrheic and norovirus positive infants were eligible for the study. These were age-matched with fecal samples from 24 non-diarrheic, norovirus negative infants, who reported at the same clinic for routine immunization and were used as controls. Two other groups; non-diarrhea norovirus positive (n = 17) and diarrhea norovirus negative infants (n = 17) were also included in the study. There were no significant differences in demographics and other baseline characteristics such as gender, age, ethnicity, weight at admission, mode of birth delivery and feeding type between the diarrheic, norovirus positive infants, and their non-diarrheic norovirus negative counterparts (Table 1). Neither was there any significant difference with the other study groups (data not shown).

**Table 1.** Demographics and other baseline characteristics of infants involved in the study and differences between diarrheic norovirus positive and non-diarrheic norovirus negative infants

Characteristic	Number of infants, (%)		
	Norovirus positives (n = 25)	Norovirus negatives (n = 24)	P value
Sex			
Male	12 (48)	12 (50)	0.7769
Female	13 (52)	12 (50)	
Age			
3.5-month old	13 (52)	11 (46)	0.1572
9-month old	12 (48)	13 (54)	
Ethnicity			
Black	25 (100)	24 (100)	1.0
Mode of birth delivery			
Natural birth	7 (28)	8 (33)	0.1381
Cesarian section	18 (72)	16 (67)	
Feeding type			
Breast milk	24 (96)	22 (92)	0.2336
Formula milk	1 (04)	2 (08)	
Average weight at admission			
3.5 month old	7.80 kg	6.88 kg	0.2446
9.0 month old	9.16 kg	8.77 kg	

**Figure 1.**
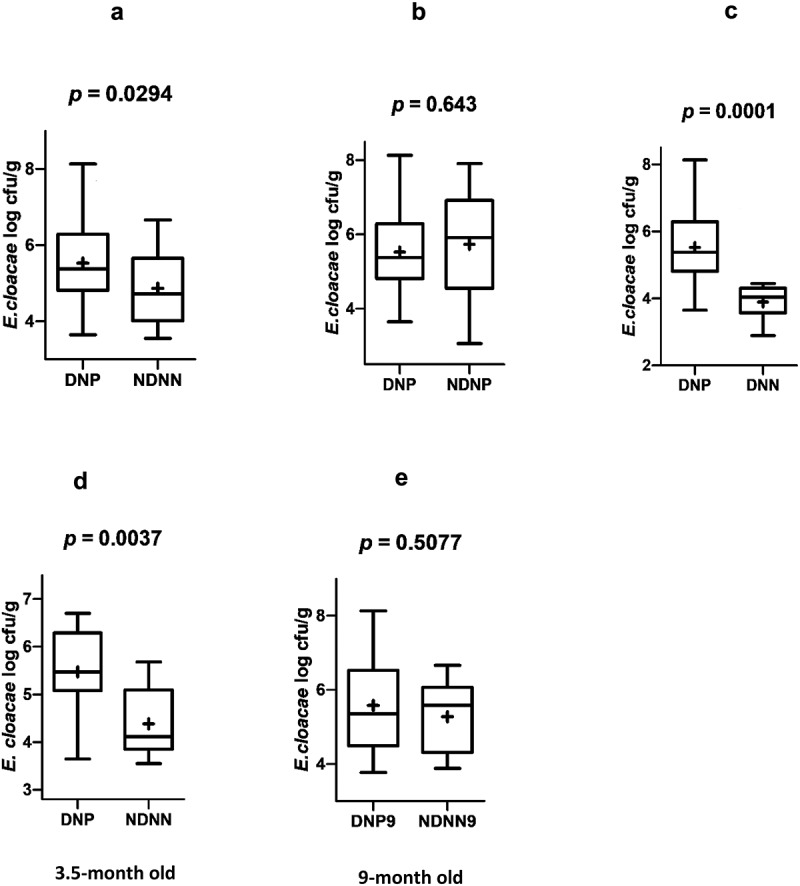
Abundance of *E. cloacae* (log CFU/g) in stool samples of the study groups. (a) Diarrheic norovirus positive (DNP) versus non-diarrheic norovirus negative (NDNN), (b) DNP versus non-diarrhea norovirus positive (NDNP), (c) DNP versus diarrhea norovirus negative (DNN), (d) 3.5-months old DNP versus 3.5-months old NDNN, (e) 9-months old DNP versus 9-months old NDNN. **^+^**represent mean of the group *E. cloacae* counts

A total of 49 fecal samples, 25 from diarrheic norovirus positive infants and the rest from their non-diarrheic norovirus negative counterparts were quantitatively assayed for *E. cloacae*. The bacterium was detected in all fecal samples of the two study groups. However, fecal samples of diarrheic norovirus positive infants contained significantly higher levels of *E. cloacae* (median 5.3796 cfu/g; IQR 1.1565 cfu/g) compared to the non-diarrheic norovirus negative infants (median 4.7201 cfu/g; IQR 1.5982 cfu/g), *p* = .0294 (Table 2, Figure 1a). When age was stratified into 3.5 and 9 months, 3.5-month old diarrheic norovirus positive infants harbored a significantly higher abundance of *E. cloacae* (median 5.4681; IQR 1.2096 cfu/g) compared to their non-diarrheic norovirus negative counterparts (median 4.1149; IQR 1.2389 cfu/g), *p*= .0037. In addition, although not statistically significantly (*p* = .5077), the median abundance of *E. cloacae* in 9-months old diarrheic norovirus positive infants was higher than those in 9-months old non-diarrheic norovirus negative infants.

The abundance of the bacterium in diarrheic norovirus positive infants was compared with those of diarrheic norovirus negative infants. Diarrheic norovirus positive infants had significantly higher counts of fecal *E. cloacae* compared to diarrheic norovirus negative infants, *p* = .0001 (Figure 1c). Comparison was also made between the abundance of the bacterium between diarrhea norovirus positive and asymptomatic norovirus positive infants, and as shown in Figure 1b, there was no significant difference in fecal *E. cloacae* counts between the two study groups (*p* = .643).

Analysis of Ct values (fecal norovirus titers) and levels of *E. cloacae* in diarrheic norovirus positive infants showed that infants with higher levels of *E. cloacae* had higher titers of fecal norovirus (lower Ct values) compared to those with lower counts of the bacterium (data not shown). However, the difference was not statistically significant.

Counts of fecal *E. cloacae* between diarrhea norovirus positive and diarrhea norovirus negative infants were used to measure the risk of norovirus diarrhea. Infants with higher counts of fecal *E. cloacae* had a significantly high risk of norovirus diarrhea compared to those with lower counts of the bacterium (odds ratio (OR) = 6.14, 95% confidence interval (CI) = 1.56–23.99, *p* = .009 (Table 3). Contribution of potential confounding factors such as sex, age, mode of delivery and infants’ feeding type were assessed in predicting *E. cloacae* counts, and the results are also shown in Table 3. The OR of age of the infant in predicting the *E. cloacae* counts in the fecal samples was 1.0 (95% CI: 0.824–1.225, *p* = .963), while sex, mode of birth delivery and average weight at recruitment were 1.71 (CI: 0.512–5. 687, *p* = .384), 1.38 (CI: 0.381–5.037, *p* = .621) and 1.11 (CI: 0.729–1.701, *p* = .616) times, respectively, likely to predict the fecal bacterial counts.

**Table 2.** Descriptive statistics of the two sets of data presented in terms of median values and interquartile range (IQR)

	Fecal E. cloacae abundance (log cfu/g)						
	Diarrheic norovirus positive infants			Non-diarrheic norovirus negative infants			
**Age group**	**n**	**Median**	**IQR**	**n**	**Median**	**IQR**	***p* value**
All ages	25	5.3796	1.1565	24	4.7201	1.5982	0.0294
3.5 months	13	5.4681	1.2096	11	4.1149	1.2389	0.0037
9 months	12	5.3533	1.7557	13	5.5801	1.4365	0.5077
	**Diarrheic norovirus positive infants**			**Non-diarrheic norovirus positive**			
**Age group**	**n**	**Median**	**IQR**	**n**	**Median**	**IQR**	***p* value**
All ages	25	5.3796	1.1565	13	5.9138	2.369

**Table 3.** Association between counts of fecal *E. cloacae* and norovirus diarrhea, sex, age, mode of birth delivery and weight of the infants at study enrollment

	Variables	Odds ratio	95% Confidence interval	p value
Norovirus diarrhea	6.14	1.560–23.991	0.009	
Sex	1.71	0.512–5. 687	0.384	
Age	1. 00	0.824–1.225	0.963	
Mode of birth delivery	1.38	0.381–5.037	0.621	
Weight at enrollment	1.11	0.729–1.701	0.616	

Only saliva from diarrheic norovirus positive infants were available for detection of HBGA and attempts to detect the antigens from both diarrheic norovirus positive and non-diarrheic norovirus infants from fecal material was unsuccessful. HBGA were assayed from 19 of the 25 diarrheic norovirus positive infants and from these, the majority (64.3%) were secretors, and the rest were non-secretors. In addition, 68.4% (13/19) of diarrheic norovirus positive infants were Lewis A^+^B^+^ and the rest were Lewis A^−^B (15.8%, 3/19), Lewis were A^+^B^−^ (10.5%, 2/19) and Lewis A^−^B^+^ (5.3%, 1/19).

Several innate risk factors including HBGA expressed by the host^4^ have been associated with norovirus infection. The current study investigated the abundance of *E. cloacae* between norovirus infected and non-infected infants, and assessed whether harboring higher counts of the bacterium is also a risk factor for norovirus diarrhea. The bacterium was detected in all fecal samples of the four study groups, indicating that colonization of the gut by this bacterium begins very early in life and is consistent with observations made by other studies.^12^ However, norovirus positive infants contained significantly higher counts of *E. cloacae* in their fecal samples compared to infants who were negative for the norovirus carriage, and this was higher whether the norovirus infection was symptomatic or asymptomatic. This is the first study to report such significant differences in abundance of *E. cloacae* between these study groups, and suggests that harboring increased levels of *E. cloacae* can be a risk factor for norovirus infection. Indeed, logistic regression analysis of *E. cloacae* counts between diarrheic norovirus positive and diarrheic norovirus negative infants indicated that harboring higher high counts of the bacterium increased the risk of diarrhea due to norovirus. Not all *E. cloacae* strains have been shown to express HBGA antigens. However, the *dna*J sequence of strains detected in the current study were 99.9% identical to *E. cloacae* subspecies cloacae ATCC 13047, which has been shown to bear H-type HBGA on its cell surface.^11^ As mentioned previously, HBGA-bearing *E. cloacae* has been shown to promote norovirus infection by binding to the virus and facilitating the productive attachment and infection of target cells.^6^ As such harboring high numbers of this bacterium in the gut increases the likelihood of more noroviruses being facilitated to attach to the target cells and cause an efficient infection.^6^

The study found that fecal *E. cloacae* counts between diarrhea norovirus positive and asymptomatic norovirus positive infants were similar. It is not known whether asymptomatic norovirus positive infants were still convalescing from norovirus diarrhea prior to their enrollment or that they were truly asymptomatic. Detection of the virus in the convalescing phase may indicate long-term viral shedding rather than asymptomatic infection.^13^ However, human studies have shown the existence of truly asymptomatic norovirus infection due to the absence of susceptible factors to symptomatic infection.^14^ The current observation suggests that higher counts fecal *E. cloacae* is a risk factor for both symptomatic and asymptomatic norovirus infection.

When age was stratified, the difference in abundance of fecal *E. cloacae* between the diarrheic norovirus positive and their non-diarrheic norovirus negative counterparts was more significant among 3.5-month olds compared to 9-month old infants. This suggests that at a younger age, infants with higher counts of the bacterium could be more prone to norovirus diarrhea than those with lower *E. cloacae* counts. As stated previously, the host HBGAs act as cell attachment factors for norovirus infection.^4^ However, some HBGA secretor negative individuals are still susceptible to norovirus infections.^15^ Moreover, some individuals are said to be weak secretors (Lewis A^+^B^+^), a phenomenon transiently observed early in infancy^16^ and shed viruses in feces lower than other HBGA groups.^17^ Collectively, this suggests that, in absence of other innate risk factors, increased abundance of HBGA-expressing bacteria such as *E. cloacae* could play a prominent role in promoting norovirus infections.

The study also found that none of the confounding factors (sex, age, mode of delivery, weight of the infants at recruitment) contributed significantly in predicting the abundance of fecal *E. cloacae* among study participants. Studies about differences in gut bacterial abundance between males and females have been inconsistent, with some reporting variations between the two^18–20^ while others have indicated none.^21^ The findings in the current study is consistent with the later and could partly explain why norovirus infections affect males and females equally.^21^ During the first two years of life, the abundance and composition of gut bacteria is highly variable and unstable.^22^ It is not surprising that the abundance of *E. cloacae* in fecal samples could be not predicted according the age of the infants.

The study observed that some of the diarrheic norovirus positive infants were non-secretors and Lewis A negative, suggesting that non-secretors are also susceptible to norovirus infection. This is inconsistent with studies that have shown that secretor negative individuals are resistant to norovirus infections.^23–25^ The observation in the current study indicates that the host’s HBGA are not the only susceptibility factor for norovirus infection. Interestingly, the non-secretor and Lewis A negative diarrheic norovirus positive infants harbored the highest counts of fecal *E. cloacae* and could suggest that in the absence of the host’s HBGA, possessing elevated levels HBGA-expressing bacteria can be a susceptibility factor for the infection.

The study had both strengths and limitations. One major strength is the use of quantitative bacterial profiling, rather than relative bacterial profiling as done in most studies, to assess its association with norovirus diarrhea. Although it has its own biases, quantitative bacterial profiling provides information about the extent of changes in species or strain abundance, and if bacterial counts differ substantially between samples, it can allow attempts to link bacterial features to quantitative data such as physiological parameters or metabolite concentrations.^26^ One major limitation of the study is the small number of samples used to evaluate the differences in abundance of *E. cloacae* between the two study groups. Larger sample size studies are required to validate the current findings. In addition, the study involved black South African infants only and it would be interesting to see *E. cloacae* counts in other ethnic groups as well, as studies have indicated variations in diversity and composition of gut bacteria (at species and strain level) among different ethnic groups. Furthermore, the qPCR assay used to quantify the *E. cloacae* accounts does not necessarily provide numbers of viable bacteria but bacterial genomic copies present in the sample.

In summary, the study has showed, for the first time, significant differences in abundance of fecal *E. cloacae* between norovirus-infected infants and their non-infected counterparts. These differences were more significant among the 3.5-month old diarrheic norovirus positive infants compared to their 9-month counterparts. The risk of developing norovirus diarrhea was significantly associated with abundance of *E. cloacae*, with those harboring higher counts of the bacterium at high risk. The confounding factors such as age, sex, weight, and mode of birth delivery of infants did not contribute significantly to the prediction of abundance of the bacterium in the infants’ fecal samples. The findings of the study suggest that the abundance of these kinds of bacteria should be considered when designing oral norovirus vaccines. Future oral norovirus vaccines could be co-administered with probiotic bacteria expressing HBGAs for maximum effectiveness.

This was case-control study that involved infants who presented at Oukasie healthcare clinic in northern Pretoria, South Africa, for gastroenteritis and routine immunization program between 2018 and 2020. Only infants of similar age (3.5 to 9 months) were recruited to the study after informed and written consent from the guardians. Infants were divided into two groups; diarrheic norovirus positive infants as cases while non-diarrheic norovirus negative infants formed the control group. In addition, two other groups; non-diarrheic norovirus positive and diarrheic norovirus negative infants were also recruited for the study. Diarrhea was defined as having at least 3 loose, watery stools within 24 hours, accompanied by at least one of the following symptoms: nausea, vomiting, abdominal pain, or high fever. Infants treated with antibiotics 3 months prior to the enrollment were excluded from the study. Ethical clearance was granted by the Research and Ethics Committee of Sefako Makgatho Health Sciences University, number SMUREC/M/277/2019: IR.

Fecal samples were collected on the day of clinical visitation for both diarrheic cases and non-diarrheic controls. Fecal specimens were collected from the infants’ diapers into sterile plastic bottles and immediately stored in −20°C freezers at the clinic. Frozen samples were then transported to the laboratory within 30 min in cooler boxes containing ice blocks, where they were stored at −80°C. A 10% weight/volume stool suspension was prepared for each of the fecal samples by weighing 100 mg of the specimen into 900 mL of sterile water. Viral RNA was extracted from 140 µL of the suspension using QIAamp Viral RNA Mini Extraction Kit (Qiagen, Hilden, Germany) as instructed by the manufacturer. A total of 50 µL of viral RNA was eluted and stored at −80°C. Fecal bacterial DNA was extracted by using QIAamp Fast DNA Stool Mini Kit (Qiagen, Hilden, Germany) as per manufacturer’s instructions. A total of 150 µL of DNA was collected and stored at – 20°C for detection of *E. cloacae*.

HNoVs were detected in fecal samples by real-time quantitative polymerase chain reaction (RT-qPCR) and using Allplex Gastrointestinal (GI)-Virus Assay Kit (Seegene, South Korea) as recommended by the manufacturer. The RT-qPCR was performed in a Bio-Rad CFX96 Real-Time System (Bio-Rad Laboratories, Hercules, California) using 8-tube PCR strips (Bio-Rad Laboratories, United Kingdom). The PCR conditions involved 20 min of reverse transcription at 55°C, 15 min of initial denaturation at 95°C, 45 cycles of denaturation at 95°C for 10 s and extension at 60°C for 30 s. The RT-qPCR results were exported and analyzed in Seegene viewer software (Seegene, South Korea). A value of cycle threshold (Ct) less than 40 was considered as norovirus-positive.^27^

The bacterium was detected in fecal DNA samples by real time qPCR using primers dnaJF4 (gac gct gat taa aga tcc atg cac) and dnaJR5 (cac ccg tat cta cgc cag cc) (this study) that target the *dna*J gene sequence of *E. cloacae* ATCC 13047, and a probe described by Pavlovic et al.^28^ In short, a 20 µL qPCR reaction mixture consisted of 2X Luna Universal qPCR Master Mix (New England BioLabs, Massachusetts, USA), 0.4 µM each of the forward and reverse primers, 0.2 µM dnaJ P, 5 µL DNA template with nuclease-free water making up the rest. The qPCR assay was done in a Bio-Rad CFX96 Real-Time System (Bio-Rad Laboratories, Hercules, California) using 8-tube PCR strips (Bio-Rad Laboratories, United Kingdom) under the following conditions: 1 min of initial denaturation at 95°C, 45 cycles of denaturation at 95°C for 15 s and extension at 60°C for 30 s.

The standard curve for *E. cloacae* was constructed by using DNA isolated from *E. cloacae* subspecies cloacae ATCC 3047. Briefly, ten-fold dilutions were made from *E. cloacae* subspecies cloacae ATCC 3047 C collected in logarithmic growth phase from *E. coli* (EC) broth (Neogen, Ayr, Scotland) culture. Each dilution was plated in triplicates on blood agar plates to determine the bacterial colony forming units (CFU). The remainder of each dilution was used to extract DNA using a QIAamp DNA Stool Mini kit (Qiagen, Hilden, Germany) as recommended by the manufacturer. The standard curve was generated by qPCR amplification of DNA isolated from 10-fold serial dilutions and plotting CT values of each dilution against their corresponding CFU.

Lewis HBGA phenotypes of the study participants were determined by a saliva-based ELISA using MAb specific to Lewis A and B antigens (DiClon Anti-Lea /Anti-Leb, Bio-Rad, Switzerland), as described previously.^29,30^ The secretor status was determined using a lectin based ELISA assay specific for Fuca1-2 Gal-R present in saliva of secretors, but not non-secretor, as done previously.^29,30^

Statistical significance of differences in baseline characteristics between the norovirus infected and non-infected infants were determined by the χ2 test. Bacterial counts (cfu/g) were transformed into log form and analyzed using Prism 8.4.3 (GraphPad Software, San Diego, USA). The Shapiro Wilk and Kolmogorov Smirnov tests were used to test the distribution of bacterial counts data sets. Descriptive statistics were presented in terms of median values and interquartile range (IQR) and displayed as box plots. Unpaired t test with Welch’s correction was used to compare the differences in abundance of *E. cloacae* between the study groups. Log cfu/g of fecal *E. cloacae* was used as a continuous variable to test the association between bacterial counts and the risk of norovirus diarrhea. Odds ratio (OR) and 95% confidence interval were estimated using logistical regression. Multiple logistical regression analysis was also used to assess the contribution of potential confounding factors in predicting *E. cloacae* counts. In all statistical analyses, a *p* value less than 0.05 was considered significant.
